# Non-equilibrium phase transition at a critical point of human blood

**DOI:** 10.1038/s41598-021-01909-9

**Published:** 2021-11-17

**Authors:** Mariusz A. Pietruszka

**Affiliations:** Faculty of Natural Sciences, Institute of Biology, Biotechnology and Environmental Protection, University of Silesia, 28 Jagiellońska St., 40032 Katowice, Poland

**Keywords:** Biological physics, Statistical physics, thermodynamics and nonlinear dynamics, Biophysics, Physiology

## Abstract

Blood is the basic medium in the existence, evolution and physiological balance of animals and represents the biochemical “Internet” of the body; at least human blood exhibit the presence of an emergent phase that is highly unusual. Homeostasis, the state of the optimal functioning of the body, is maintained in living organisms by many chemical and physical conditions, particularly temperature. However, no regulatory mechanism has been identified that has led to a predetermined (molecularly encoded) optimal, individually variable, very specific temperature of around 36 °C. Additionally, the homeostatic temperature range, which is kept within predetermined limits, is merely an empirical fact. In the following, I will show that the *reference temperature* that is necessary to achieve homeostasis can be established, and a preset *homeostatic range* can be determined, using an original experimental method and refined tools of mathematical physics related to the nonlinear measures of the complexity of human blood. Moreover, signatures of a macroscopic coherent state in a non-equilibrium system at a critical temperature are obtained.

## Introduction

Quantum dynamics underlies macroscopic systems, such as crystals, ferromagnets or superconductors which exhibit some kind of ordering. Furthermore, ordering in biological systems appears to be dynamically generated out of the microscopic scale of fluctuating quantum components^1^, however, no temperature-induced phase transition has yet been discovered. After detecting a scale-free behaviour in the ion fluxes^2^ of an isolated droplet of human blood^3^, a non-equilibrium phase transition was revealed^4^ at a physiological (optimum) temperature ${T}_{c}=36$ °C. Here, I show that there is an emergent macroscopic coherent state^5–7^, which is reflected by a point-like attractor in the phase space where the optimal dynamical range^8^ is two orders of magnitude greater than that outside of the critical region. Simultaneously, the computed dynamic entropy reached a deep minimum, while the spectral signature indicated criticality. This picture is reinforced by the different nonlinear complexity metrics that also had a distinct extreme that identified the critical point. Moreover, the *molecular coding of the optimal temperature of life* as a benchmark for homeostasis was identified through direct observation of spontaneous electric currents (charged avalanches^9^) that peaked at ${T}_{c}$ of metabolically active red blood cells providing evidence for the existence of cooperative phenomena (collective excitations) that multicellular systems should benefit from. Because of the resonant transmission at the critical point, a zero-bias conductivity peak was observed, while the calculated information entropy^10^ equalled only one bit. These biologically relevant results broaden our understanding of non-equilibrium phase transitions into living systems and elucidate a physical aspect of homeostasis^11^ since all of the regulatory mechanisms are unclear and incomplete unless an optimal (reference) temperature has been established.

From a physical point of view, a description of living biological systems should take into account the dynamics of the biological processes and the fact that they are open thermodynamic systems, i.e., they interact and exchange heat, nutrients and information with the outside world and obey the rules of non-equilibrium thermodynamics^12^; this dynamics is intrinsically dissipative. However, dissipativity, which is typical of the biological systems, was shown to be the macroscopic manifestation of a microscopic invariance law^13^, while the spontaneous breakdown of symmetry, via the Anderson-Higgs-Kibble mechanism, manifested itself in a self-focusing propagation for the electromagnetic field inside the biological systems^14^.

The thermodynamic state of equilibrium of isolated systems can be characterised by the minimum Helmholtz free energy, which is defined by the internal energy, absolute temperature and entropy. However, in a living biological system, which is constantly exchanging energy and matter with the environment, new types of dynamic states of matter, which are called dissipative structures, can become the source of order^15^. These structures have a coherent, collective nature that leads to new macroscopic manifestations^16^. [Coherence determines properties at a given space–time point when they are known at another, such as phase and amplitude of coherent waves, i.e., two waves are coherent if they have a constant relative phase]. In this context, the occurrence of a minimum in dynamic entropy at about 36.6(5) °C of human blood^4^ and at 25.9(5) °C in a population of tobacco pollen tubes^7^ that indicated the existence of (correlated) temperature-controlled processes at these particular temperatures could have been anticipated. Moreover, it turned out that in the mutual interaction of the ionic currents of cells an individually variable optimal temperature is coded^4,7^, which led to a well-founded assumption about the molecular coding of these characteristic temperatures.

Chemical potential is the energy that can be absorbed or released as a result of changing the number of particles. Similar to the other thermodynamic potentials, the abrupt changes of the chemical potential locate critical temperatures in the studied system of condensed matter in the phase transitions^17–21^. Moreover, it has been found that these phase transitions can be detected by some kind of proximity effect using the contact electrode method^22,23^. A similar “contact” method was proposed to determine the level of the chemical potential (redox) for the ion oscillations of individual (living) cells using the n-type semiconductor-electrolyte interface^2^. This method made it possible to localise the characteristic temperatures of both the growing pollen tubes^3,7^ and peripheral human blood^4^.

To investigate the possibility of a critical point in a single droplet of living human blood, high-precision (μV) voltage measurements (in number of 169, sampling rate 4.1 Hz) were taken, each collected in a 20-min. time series, which is a sequence of observations recorded at a succession of time intervals ($N=5000$), in an adiabatic Faraday cage using a semiconductor measuring device^2^. In this unique non-invasive experiment, the sample was intact or under the influence of a magnetic field ($B\ne 0$) from a toroidal magnet that was placed on top of a semiconductor plate. Then, similar advanced statistical mechanics / programming tools in R^24^ (so-called R code) were used to analyse the detected signal, which were used to observe the gravitational waves^25^. However, this time, they were used to analyse the electromotive force (EMF) time series that leads to the detection of the synchronized (phase coherent) ion plasma waves that can form a macroscopic coherent state in human blood. Details of the measurement procedures are provided in “Materials and methods” section.

## Results

### Application of the time-series regularity metrics to the ion flux data

In biological activity, (metabolic) energy supply leads to the establishment of organisation, Prigogine’s dissipative structures^16,26^. Prigogine's principle^26^ of minimum entropy production (least entropy formation), which is also considered to be the fourth law of thermodynamics, states that the systems in the vicinity of the equilibrium (steady) state go through (non-equilibrium) states in which the production of entropy is the lowest. The principle of minimum entropy production says that the steady state of an irreversible process, i.e., the state in which the thermodynamic variables are independent of the time, is characterized by a minimum value of the rate of entropy production^27^. Dynamic entropy—a measure of regularity^28,29^—should be able to quantify the complexity of any underlying structure in discrete time signals. The approximate entropy, ApEn, which is a mathematical formula^30^ that quantifies regularity over time-series data, proved its ability to distinguish different system dynamic, e.g. Refs.^4,7^.

**Figure 1:**
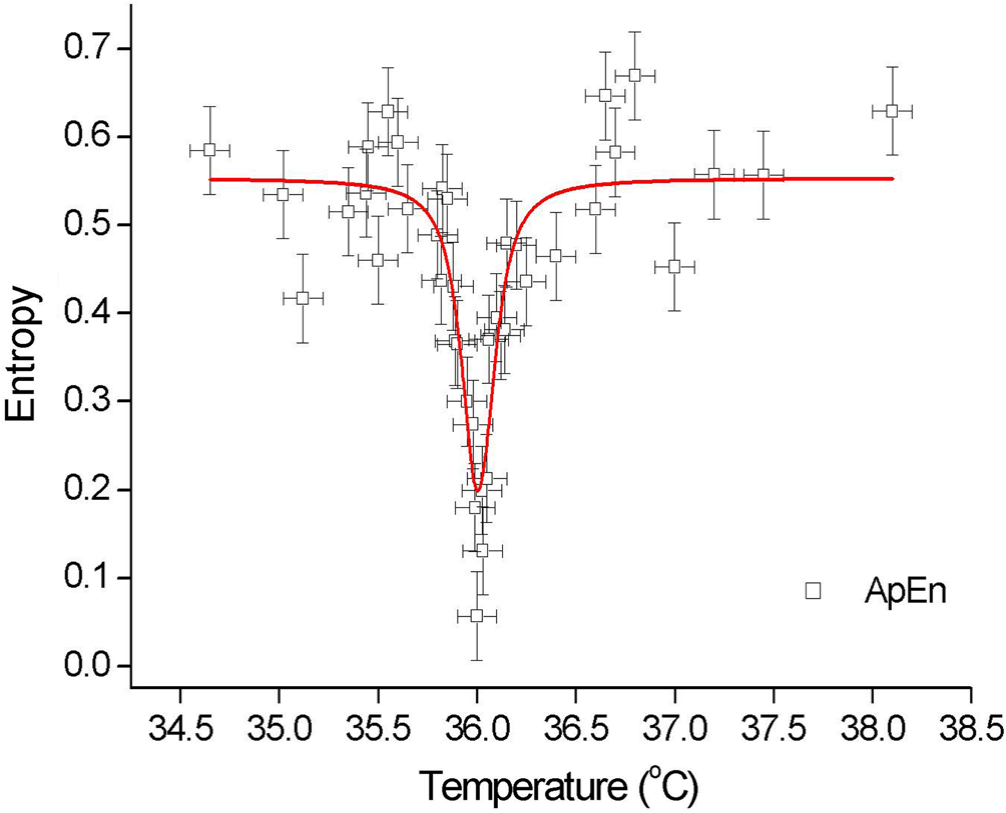
Dynamic entropy as a function of the temperature of human blood. Approximate entropy (ApEn squares) was *calculated* (each point separately) for the *empirical* electromotive force time series that was induced by the ionic fluxes of an isolated (intact) droplet of human blood. Indicated critical temperature ${T}_{c}=36.01(1)$ °C and division into three phases: subcritical (time dependent, irreversible), critical (“timeless”, quasi-reversible, steady state) and supercritical (time dependent, irreversible) showing the non-equilibrium phase transition. Determination coefficient for the experimental data that was re-calculated in R code (series of 5000 time points for each temperature at 40 different temperatures; sampling rate 4.1 Hz) fitted to the Lorentz resonance curve that equalled R^2^ = 0.82635, χ^2^ = 0.004, half-width w_1/2_ = 0.17(2) °C, $\Delta T=0.1$ °C; $\Delta S=$ 0.05—estimated from the difference $max\left(|{S}_{a}-{S}_{s}|\right)$ between the calculated approximate (${S}_{a}$) and sample (${S}_{s}$) entropy. The errors are represented by the drone-like objects in the plot.

The dynamic entropy, $S$, which was calculated in the R programming language^24^ at different temperatures and then fitted to the Lorentz resonance curve is presented in Fig. 1 (see also Supplementary Information Table S1). In Fig. 1, the system changed from a time-dependent (irreversible) state at low temperatures to a time-independent (quasi-reversible) state at the critical temperature, ${T}_{c}$, back to a time-dependent (irreversible) state at high temperatures. For steady states close enough to the critical point, the entropy production reaches its minimum (minimum $S$ production theorem^27^). The time-dependent states have a higher production of entropy, which is an experimental manifestation of the statement that only irreversible processes contribute to the generation of entropy.

**Figure 2:**
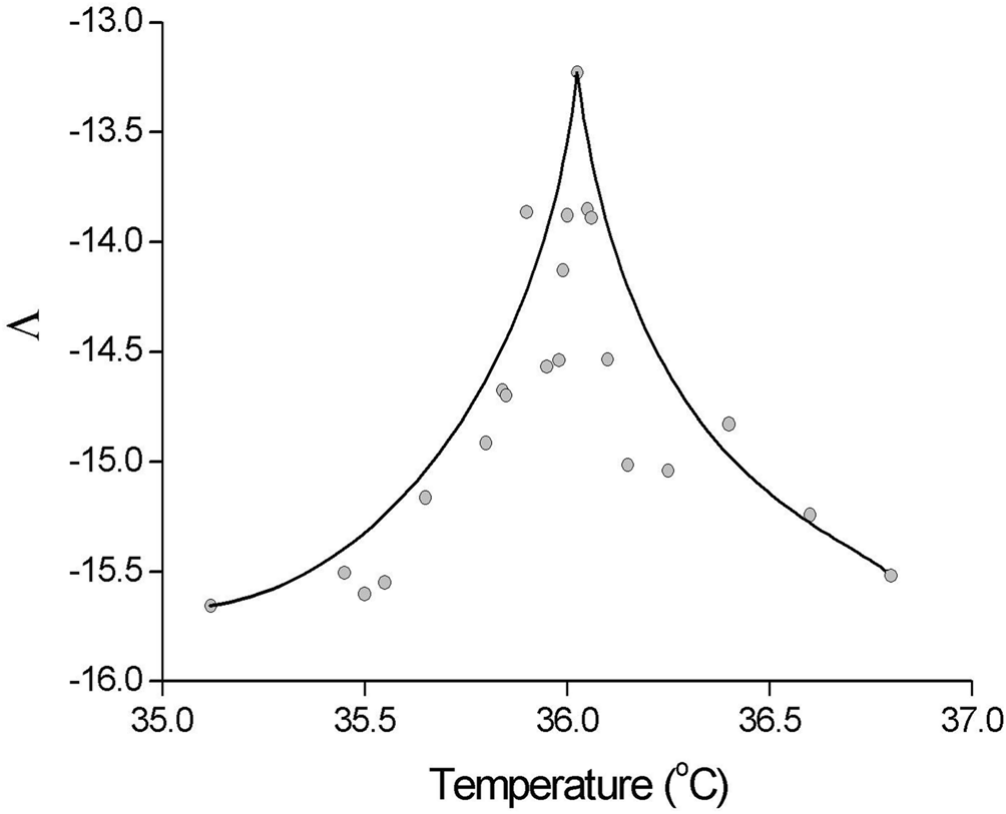
Dynamic range of the maximal Lyapunov exponent (Λ) as a function of temperature at criticality. Both sides interpolated separately by cubic splines. Compare with theoretical predictions in Fig. 2d in Ref.^8^.

The temperature-induced evolution of the largest Lyapunov^31^ exponent (which gives a measure of the total predictability of a system; negative Lyapunov exponents are characteristic of dissipative systems), which is shown as $\Lambda /T$, is illustrated in Fig. 2. As quantified in Fig. 2, $\Lambda /T$ at low temperatures shows a power law increase to a sharp maximum at ${T}_{c}$, and a steep decrease above this temperature, thereby exhibiting a dynamic range of about two to three orders of magnitude. This broad dynamic range is a collective phenomenon—compare with the theoretically predicted values presented in Fig. 2d in Ref. ^8^. It is found near the phase transitions, in the study of complex systems^8^. Here, I give a clear empirical example of a biologically relevant quantity that is optimised at criticality, where the dynamic *range* of $\Lambda$ is maximised at the critical point of the non-equilibrium phase transition. This can only be met if the spontaneous activity of living matter^2^ corresponds to a critical process (critical avalanches^9^).

**Figure 3:**
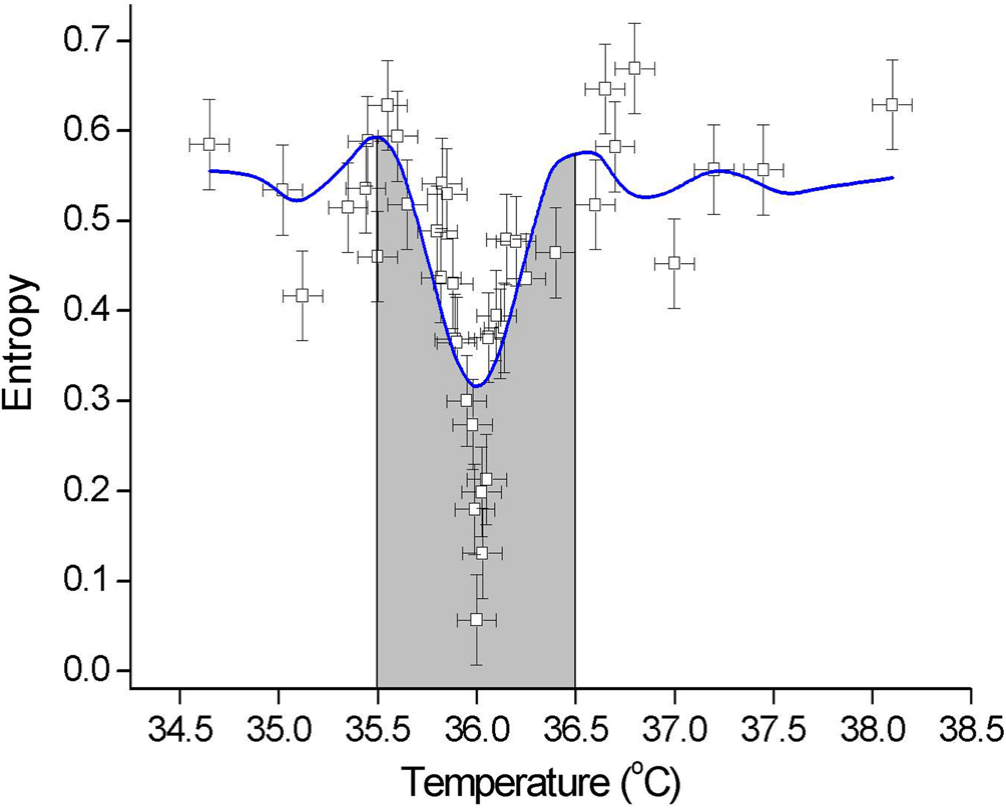
Dynamic entropy as a function of temperature. Approximate entropy (ApEn) data interpolated using the sinc function. The shading represents the attractor basin, which can be interpreted as a dynamic superconducting energy gap 1 K (0.0001 eV) wide with a *dynamic* Majorana quasi-particle peak in the centre. Fit parameters: coefficient of determination R^2^ = 0.6259 (χ^2^ = 0.007), y_0_ = 0.54(2), a = − 0.23(3), b = 8.9(9) to $y={y}_{0}+\frac{a\mathrm{sin}\left(bx\right)}{x}.$

In Fig. 3 the approximate entropy data as a function of temperature is interpolated by the sinc function (Note that the sinc function is the Fourier transform of the rectangular function—which in the limit is the Dirac delta function). Based on the BCS formula (${2\Delta =3.53{ k}_{B}T}_{c} )$ that the band gap at 36 °C (309.15 K) equals $1.76{ k}_{B}{T}_{c} \approx 0.05$ eV and the approximate gap width in Fig. 3 is 1 K (0.0001 eV), the effective mass of the charge carriers is of the order of 2 × 10^3^ m_e_, i.e., proton mass. It seems that fine tuning the critical temperature can be established—in analogy to the Majorana quasi-particle^32^ (note zero-bias peak and splitting as a signature of Majorana quasi-particles) in superconducting matter—by a symmetry-protected (dynamic) topological state in a system, thus maintaining homeostasis. Accordingly, a symmetry-protected topological state can stabilise the critical temperature of a system within the ~ 1 K interval (according to our daily life experience—outside this ± 0.5 °C range, medical intervention is usually expected), thereby providing a sophisticated mechanism for selecting and maintaining the optimal life temperature.

**Figure 4:**
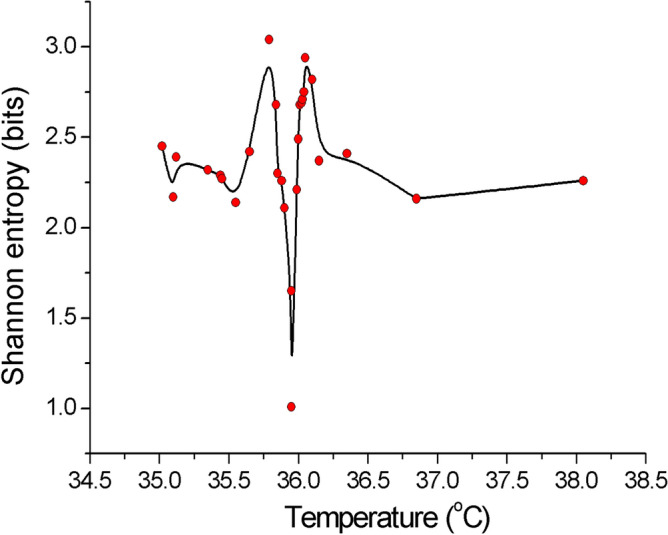
Dynamic Shannon entropy as a function of temperature in a binary representation. Calculated data points (red) were interpolated by B-spline. Note that the lowest value that was obtained at ${T}_{c}$ corresponds to one bit.

Shannon informational entropy^10^ tells what is the minimum number of bits that is required to encode information (message) in a binary form. The calculated dynamic information entropy shown in Fig. 4 partially recreates the previous results, but with a remarkable outcome: one bit was enough to describe the dynamics of the system at criticality. However, this one (q)bit can encode $|0>$ or $|1>$ state of the system (or any other point on Bloch sphere), corresponding to the observation of spontaneous discharges at a critical temperature of human blood, weighted by the number ${N}_{+}$ and ${N}_{-}$ of populations involved in coherent sub-dynamics, Figs. S1-3e. The long-range cooperative interaction between live erythrocytes can be responsible for the non-vanishing electric polarisation field; thus the order parameter $\lambda =\frac{{N}_{+}-{N}_{-}}{N}$ can characterise the asymmetric vacuum of the system and consequently can be assumed to be the relevant order parameter in the sense of statistical mechanics.

The results presented above, which converge to the same physiological temperature, are supported by Table S1 and Figs. S1-3 in the Supplementary Information, where the calculated complexity metrics are shown. There (Figs. S1-3), apart from the raw (a) and detrended (b) data the Hilbert transform (c) and the Hilbert-Huang envelope (d) in the Hilbert spectrum shows the resonance in the vibration signal, while the histogram (e) enabled the entropy of information^10^ to be calculated. The autocorrelation function (f) and the mean mutual information (g) together with the calculated embedment dimension (h) enabled us to recreate (according to Takens' theorem) the phase space trajectory, which transformed from a highly symmetrical (isotropic) state, Fig. S3 (i), into a state with lower dimensions, Figs. S1-2 (i). Note that in the figures of Figs. S1-2 (f), a linear suppression of the autocorrelation function can be observed, which means that near the critical point, there are long-range correlations (compare with fig. 7 in Ref.^15^ for a critical behaviour), which is a characteristic feature of the phase transition. It should also be noted that the electromotive force already exhibits a visually ordered feature in raw/detrended data at physiological temperature, while an apparently disordered pattern is observed outside of it.

With reference to Fig. S2 (d), the theory of phase transitions requires that the additional energy supplied causes the excitation critical value to be exceeded: If the system is in a critical state, a small power supply can trigger off an excitation. Thus, the slightly fuzzy limit cycle shown in Fig. S2 (i) may also be sensitive to weak external fields, as it was shown in Fig. S3 (i). Moreover, if the coherent state is already excited but not to its ultimate value, the energy input will increase the amplitude, as in Fig. S2 (d) for 850 – 900 s interval.

## Discussion

The spontaneous breakdown of the symmetry is not conditioned by an external stimulus. At ${T}_{c}$, a continuous (phase) space rotation is spontaneously broken. The Goldstone theorem then predicts the existence of massless Nambu-Goldstone (NG) boson particles^1^. These quanta are of a dynamical origin—they are not found in the symmetric or normal phase (beyond ${T}_{c}$); they are (phase coherent) collective modes. However, this is the ordering that is generated by the dynamical interactions among the atoms and molecules, which are necessarily quantum interactions^1^. The presence of the condensation of the NG bosons sustains the long-range ordering correlation at ${T}_{c}$. It was found that the symmetry that gets broken is the rotational symmetry of the electrical dipoles of the water molecules^33^ and the NG modes are the vibrational dipole wave quanta^34^ or plasmons-polaritons^35^. According to Ref.^1^, classical statistical mechanics and short-range forces in molecular biology do not seem to be fully adequate tools. Therefore, it is necessary to complement them with a further step to take into account the underlying dynamic quantum features. Needless to say, an appropriate simulation of the microscopic mechanism (and a “homeostatic potential”) of the above correlation has not yet been formulated, though it can be connected with water temperature anomalies at and near 36 °C, see fig. 1 in Ref.^36^.

The cytoskeleton is a system of filaments that is present in the cytoplasm of eukaryotic cells. Microtubules (MTs) are cylindrical protein structures that make up the major part of the cytoskeleton in all eukaryotic cells. They have the form of empty tubes with walls made of tubulin. By using the quantum field formalism^14^ their inner diameter is computed to be of about 14.6 nm, nearly fitting the observed value of about 15 nm. The basic unit in microtubules is α-tubulin, a heterodimer approximately 8 × 10^–9^ m = 8 nm in length, composed of two protein monomers (α and β tubulin). It can make transitions between two polar orientation states corresponding to different electric dipole moments along the tube axis. Tubulin dimmers, packed together with proteins, are arranged spirally in MT with a diameter of 25 nm. Each monomer has a molecular weight m = 50–55 kDa ≈ 91.300 × 10^–27^ kg. De Broglie wavelength $\lambda =h/\sqrt{3m{k}_{B}T}$ = 6 × 10^–4^ nm at 310 K, which means that at this classical / quantum boundary for tubulin subunits, some collective processes may arise^37^, especially that on the basis of the quantum simulations of the ion passing through the voltage-dependent ion channel, it was found^38^ that the ion was delocalized while moving through the channel, and that the ion wave oscillates at a very high frequency (time in [fs]) and transmits energy to surrounding proteins.

Life has evolved in such a way as to be able to make the best use of the physics of our universe, including quantum physics. Nobel Laureate Anton Zeilinger stated that “someday we hope we can do these experiments, in quantum superposition, with living things including the saline solution” (cf. Ref.^39^). Apparently, this article presents experiments with these “living things” (red blood cells) immersed in saline, thus obtaining the signatures of a macroscopic coherent state in a non-equilibrium system at a critical temperature. Another question for in-depth research that remains is whether the observed phenomenon was of a purely classical origin or was caused by quantum principles^40^, which are capable of producing coherent charge waves^35^ in a system; however, it seems that their macroscopic properties cannot be explained without recourse to the underlying quantum dynamics^1^. The barely discernible traces in charge transfer offer hope for a better understanding of the collective excitations (that necessarily contain essential non-linear features) in living matter—nature, rather than avoiding dissipation in system-bath interactions, specifically exploits it to direct energy transfer^41^. Moreover, the unique physical properties of the quantum coherent nano-molecular water clusters that enable life at the cellular level^42^ can be compared with the results that were obtained in this research ($B\ne 0$, Fig. S2), which have practical implications for astronauts who would likely need a weak magnetic field in a spacecraft in order to survive.

The question arises, is it possible to use the presented experimental protocol to calculate other points of the non-equilibrium phase diagram of human blood? Although the computation of the critical point is always representative because of the phenomenological richness inherited from the theory of critical phenomena, it is useful to know whether it is possible to obtain binodal or spinodal curves of this system. Recent advances in obtaining non-equilibrium phase diagrams^43,44^, and theoretically defining and predicting these non-equilibrium phases and their time-evolving phase diagrams given the underlying molecular interactions^45^, show that the knowledge of these lines in the phase diagram of a substance allows us to explain non-equilibrium transitions^46^ such as gelling or vitrification (depending on the nature of the interaction forces between pairs of particles). Knowledge of these phases (by taking into account, for example, additionally the dilution factor of the sample), one could argue a little about the effects of blood clotting and related rheological measurements, which in turn provide valuable medical information.

When a system is out of balance, there is no single temperature, in other words, there can be multiple temperatures in a non-equilibrium system. We can speak of a temperature in a non-equilibrium system only when such a system is locally in thermal equilibrium^12^. The non-equilibrium system does not have one specific temperature because it is not in equilibrium. However, we can determine the temperature at any time as long as the system is locally at equilibrium. In the context of the proposed experiment, we can interpret the temperature as a slowly changing (variable) average ambient temperature in contact with the sample at any point in the time series. The thermal fluctuations create a very specific (critical) temperature (when resonance conditions are met) at which the phase transition takes place, see Fig. S2. It is easy to imagine that the transition can be overlooked assuming too large temperature intervals between consecutive measurements. Hence, temperature fluctuations help to some extent to establish the correct (critical) temperature.

## Conclusions

In the phase transition, all thermodynamic observables have a rapid change of character which determines the critical temperature^5^. At the critical temperature controlling the transition between the various phases, “normal” molecules coexist in equilibrium with “condensed” molecules. Here I showed the existence of a non-equilibrium phase transition at the critical temperature of isolated human (and possibly other mammals) blood. Moreover, this research showed that although the dynamics of an organism is encoded in its molecular basis, non-equilibrium statistical physics (thermodynamics) and information theory are fundamental to understanding it at the microscopic level, not to mention the fact that quantum field dynamics is not confined to the microscopic world^1^. Ion “superfluidity” (frictionless ion flux, non-dissipative—which should exceed that arising from diffusion or active transport, with no affinities, no gradients of temperature and no gradients of chemical potential) or ion plasma oscillations, when considered in the context of evolutionary fitness, can produce an evolutionarily favourable survival mechanism in biological systems at the optimum temperature that is represented by the macroscopic coherent state constructed by the condensation of the lowest energy quanta associated with long range correlations or (nonlinear) collective excitations. Furthermore, in 1932, Joseph Barcroft a British physiologist, was the first to say that higher brain function required the most stable internal (temperature_MP_) conditions. The aforementioned collective phenomena occur at room temperature (here: physiological) and higher temperatures, e.g. in crystal and magnets, but also in the condensed matter of *living* systems, which are governed by the laws of non-equilibrium thermodynamics. Schrodinger's observation^47^ that the study of life will bring new physical problems that have not yet been studied by physicists seems to be looming in the field of biological physics, and this would allow a true new frontier in physics to emerge.

## Materials and methods

### Measuring device

The measurement apparatus (Supplementary Information Fig. S6) consisted an external polystyrene thermostat (26 × 31 × 24 cm) that had been completely darkened with black cardboard and an internal thermally insulated polystyrene measuring chamber (638 cm^3^) that was coated with an aluminium-grounded Faraday cage containing a semiconductor-solute interface^2,3^. The temperature control system consisted of an integrated control circuit (Fig. S7) and a 1 W heater (ceramic resistor). The temperature of the system was stabilised for about 60 min. Experiments were carried out in the geomagnetic field of 50 μT. Experiments were also performed in presence of external (constant) magnetic fields up to 60 mT.

### Samples

The measurements were taken on the peripheral blood of *Homo sapiens* (female, 32), which had been taken from a finger immediately before the experiment. Under sterile conditions, 40 µl of the blood (taken on an empty stomach) that had been obtained was diluted 1:1 in 0.9% sodium chloride (NaCl). The conductivity measurements, which were taken using a CC-105 conductivity metre (Elmetron CC-105, Poland), revealed that the conductance of 40 μl 0.9% NaCl (5.0(1) S/cm) plus 40 μl of the blood that had been diluted (in order to avoid sample aggregation) in 5 ml of demineralised water (0.004(1) S/cm) at a peak value of 0.356(5) S/cm at close to 36 °C (Fig. S5) was required to ensure the proper (electrolytic) conditions during the measurements.

*Comment* Upon dilution of the blood, it can be expected that the true nature of the blood will change to some extent (e.g., ATP may be released from red blood cells during processing and the fluctuation amplitude of red blood cells was found to decrease^48^), but the most important empirical result, namely the value of the physiological temperature, seems to have remained intact. Indeed, the time data series collected in the experiment depends on the state of the system and the protocol for preparing the experiment, whereas the non-equilibrium measures of the system depend on these initial conditions. However, interestingly, the critical point obtained from these varying conditions seems independent of these conditions, see also Figs. 1, 2, 3 and 4 where the “scattered” data tapers to a minimum at ${T}_{c}$.

### Electromotive force measurements

Next, 40 µl of the blood electrolytic solution was downloaded, stirred in an ELPIN + type 357 water bath shaker at a speed of 130 rpm for 5 min. and transferred (Fig. S8) onto a photovoltaic semiconductor plate^2^ (n–p, phosphorus–boron, junction on Si crystal); see Ref.^2^ for the measurement principles. Each measurement was taken in the dark chamber for 20 min at a 4.1 Hz sampling. The external conditions were a temperature in the range of 34.5–38.5 °C and 45–65% humidity, in order to avoid sample evaporation during experiment. In the physiological temperature range, the DC voltage (EMF can be measured as an open circuit potential difference or voltage which can drive an electric current if the external circuit is connected to the terminals) measurements (digital filter on) were taken, which captured a mean field of the collective of cells, at a 4.1 Hz sampling using a DMM 4040 6–1/2 Digit Precision Multimeter from Tektronix, Inc. and then recorded as a 20-min. time series ($N=5000$) on external media. Similar measurements were taken in the constant magnetic field (Fig. S9) with ferrite or neodymium magnets.

### Data analysis

The series of time data ($N=5000$), which were collected at each temperature using this non- invasive solute–semiconductor interface technique, were detrended and analysed using a program that was written in R^24^. The nonlinear statistical metrics, namely the Hurst exponent, the largest Lyapunov exponent and the entropy of an experimental time series for the detected ion currents were quantitatively evaluated (Table S1). The (corrected R/S) Hurst exponent^49^ is used to measure the long-term memory of a time series. It refers to the autocorrelation of a time series and the rate at which it decreases with an increasing delay between the pairs of values^50^. The maximum Lyapunov exponent (Λ) is the rate of the exponential separation with the time of the initially close trajectories in the phase space. It describes the speed of the convergence or divergence of the trajectories in each dimension of the attractor and estimates the amount of chaos in a system^51^. Quantitatively, two trajectories in phase space with initial separation vector $\delta {{\varvec{Z}}}_{0}$ diverge at a rate given by $|\delta {\varvec{Z}}\left(t\right)|\approx {e}^{\lambda t}\delta {{\varvec{Z}}}_{0}$ where λ is the Lyapunov exponent. The dynamic entropy (ApEn^30^, Sample^52^) quantifies the size of the fluctuation regularity in a time series (Table S1). A low entropy value indicates that a time series is deterministic, while a high value indicates its randomness. The Shannon information entropy^10^

$$H\left(X\right)=-\sum_{i=1}^{N}p\left({x}_{i}\right){\mathrm{log}}_{b}p\left({x}_{i}\right)$$

was calculated in R ($b=2$) from the histogram data, e.g., Fig. S10, at physiological temperatures using the entropy.ChaoShen() procedure^53^ and interpolated with the cubic curves (Fig. 4). The phase space was reconstructed on the basis of the original time series by virtue of Takens’ theorem^54^. The power spectral density (PSD), which was calculated using the Welch method^55^ and shadowed by the results of the discrete Fourier transform (light grey), manifested a $1/{f}^{\beta }$ noise behaviour for about three decades of the frequencies (Fig. S4). The spectral signature $\beta$ was determined from a linear slope.

The measurements were feasible due to the subtle effects (bending of the energy bands) that were taking place at the semiconductor-liquid interface^56^.

### Ethical approval

There is a study-specific procedure and approval (KEUS/0.91R/02/2021) of the Scientific Research Ethics Committee of the University of Silesia in Katowice, Poland and the informed consent was obtained from the human participant. All methods were carried out in accordance with relevant guidelines and regulations.

## Supplementary Information

Supplementary Information.

## Data Availability

All data are available in the main text or the supplementary materials.

## References

[1] Blasone M, Jizba P, Vitiello G (2011). Quantum Field Theory and Its Macroscopic Manifestations. Boson Condensation, Ordered Patterns and Topological Defects.

[2] Pietruszka M, Olszewska M, Machura L, Rówiński E (2018). Single measurement detection of individual cell ionic oscillations using an n-type semiconductor—electrolyte interface. Sci. Rep..

[3] Pietruszka M, Olszewska M (2020). Extracellular ionic fluxes suggest the basis for cellular life at the 1/*f* ridge of extended criticality. Eur. Biophys. J..

[4] Pietruszka MA (2021). Dynamic entropy of human blood. Sci. Rep..

[5] Annett JF (2004). Superconductivity, Superfluids and Condensates.

[6] Vitiello G (2012). Fractals, coherent states and self-similarity induced noncommutative geometry. Phys. Lett. A.

[7] Pietruszka MA (2021). Application of time-series regularity metrics to ion flux data from a population of pollen tubes. Commun. Integr. Biol..

[8] Kinouchi O, Copelli M (2006). Optimal dynamical range of excitable networks at criticality. Nat. Phys..

[9] Bak P (1997). How Nature Works: The Science of Self-Organized Criticality.

[10] Shannon CE (1948). A mathematical theory of communication. Bell System Tech. J..

[11] Cannon WB (1932). The Wisdom of the Body.

[12] Keizer J (1987). Statistical Thermodynamics of Nonequilibrium Processes.

[13] Del Giudice E, Doglia S, Milani M, Vitiello G (1985). A quantum field theoretical approach to the collective behaviour of biological systems. Nucl. Phys. B.

[14] Del Giudice E, Doglia S, Milani M, Vitiello G (1986). (1986) Electromagnetic field and spontaneous symmetry breaking in biological matter. Nucl. Phys. B.

[15] Prigogine I (1978). Time, structure, and fluctuations. Science.

[16] Fröhlich H, Fröhlich H, Kremer F (1983). Coherence in biology. Coherent Excitations in Biological Systems.

[17] Matlak M, Pietruszka M (2000). Critical behaviour of the chemical potential at phase transitions. Phys. B.

[18] Matlak M, Pietruszka M, Gosławska E, Grabiec B, Eid K (1999). On the new universal possibility to detect phase transitions in correlated electron systems. Phase Trans..

[19] Matlak M, Pietruszka M, Rówiński E (2000). Experimental method to detect phase transitions via the chemical potential. Phys. Rev. B.

[20] van der Marel D (2004). Electrons and bursting waterworks. Phys. Status Solidi (b).

[21] Matlak M, Pietruszka M (2001). Comparative study of the specific heat and chemical potential at phase transitions. Solid State Commun..

[22] Matlak M, Pietruszka M (2004). Phase transitions detection by means of a contact electrode. Phys. Status Solidi (b).

[23] Matlak M, Molak A, Pietruszka M (2004). Chemical potential induced phase transitions. Phys. Status Solidi (b).

[24] R Core Team. R: A language and environment for statistical computing. R Foundation for Statistical Computing, Vienna, Austria (2020). http://www.R-project.org/

[25] Abbott BP (2016). Observation of gravitational waves from a binary black hole merger. Phys. Rev. Lett..

[26] Glansdorff P, Prigogine I (1971). Thermodynamic Theory of Structure and Fluctuations.

[27] Klein MJ, Meijer PHE (1954). Principle of minimum entropy production. Phys. Rev..

[28] Wehrl A (1978). General properties of entropy. Rev. Mod. Phys..

[29] Bandt C, Pompe B (2002). Permutation entropy: a natural complexity measure for time series. Phys. Rev. Lett..

[30] Pincus SM (1991). Approximate entropy as a measure of system complexity. Proc. Natl. Acad. Sci. USA.

[31] Wolf A, Swift JB, Swinney HL, Vastano JA (1985). Determining Lyapunov exponents from a time series. Phys..

[32] Sato M (2012). Majorana fermions in topological superconductors with spin-orbit interaction. J. Phys. Conf. Ser..

[33] Jibu M, Pribram KH, Yasue K (1996). From conscious experience to memory storage and retrieval: The role of quantum brain dynamics and boson condensation of evanescent photons. Int. J. Mod. Phys. B.

[34] Del Giudice E, Mańka R, Milani M, Vitiello G (1988). Non-constant order parameter and vacuum evolution. Phys. Lett. B.

[35] Jacak JE, Jacak WA (2020). New wave-type mechanism of saltatory conduction in myelinated axons and micro-saltatory conduction in C fibres. Eur. Biophys. J..

[36] Pietruszka M, Lipowczan M (2017). Are water temperature anomalies conjugated to brain functions in microtubules?. NeuroQuantology.

[37] Pietruszka M, Lipowczan M (2014). Check sum computing in doubly frustrated microtubule clusters. NeuroQuantology.

[38] Summhammer J, Sulyok G, Bernroider G (2018). Quantum dynamics and non-local effects behind ion transition states during permeation in membrane channel proteins. Entropy.

[39] Abbott D, Davies PCW, Pati AK (2008). Quantum aspects of life.

[40] Pines D (1999). Elementary Excitations in Solids.

[41] Cao J, Cogdell RJ, Coker DF (2020). Quantum biology revisited. Sci. Adv..

[42] Davidson RM, Lauritzen A, Seneff S (2013). Biological water dynamics and entropy: A biophysical origin of cancer and other diseases. Entropy.

[43] Guo H, Ramakrishnan S, Harden JL, Leheny RL (2011). Gel formation and aging in weakly attractive nanocolloid suspensions at intermediate concentrations. J. Chem. Phys..

[44] Olais-Govea JM, López-Flores L, Medina-Noyola M (2015). Non-equilibrium theory of arrested spinodal decomposition. J. Chem. Phys..

[45] Medina-Noyola M, Zepeda-Lopez JB (2021). Waiting-time dependent non-equilibrium phase diagram of simple glass- and gelforming liquids. J. Chem. Phys..

[46] Ramirez-Gonzalez P, Medina-Noyola M (2010). Aging of homogeneously quenched colloidal glass-forming liquid. Phys. Rev. E.

[47] Schrödinger E (1962). What is Life? The Physical Aspect to the Living Cell.

[48] Betz T, Lenz M, Joanny J-F, Sykes C (2009). ATP-dependent mechanics of red blood cells. PNAS.

[49] Hurst HE (1951). Long-term storage capacity of reservoirs. Trans. Am. Soc. Civil Eng..

[50] Weron R (2002). Estimating long range dependence: finite sample properties and confidence intervals. Phys. A.

[51] Hegger R, Kantz H, Schreiber T (2002). Practical implementation of nonlinear time series methods: the TISEAN package. Chaos.

[52] Yentes JM, Hunt N, Schmid KK (2012). The appropriate use of approximate entropy and sample entropy with short data sets. Ann. Biomed. Eng..

[53] Chao A, Shen T-J (2003). Nonparametric estimation of Shannon's index of diversity when there are unseen species in sample. Environ. Ecol. Stat..

[54] Takens, F. Detecting strange attractors in turbulence. Lect. Notes Math. 366–381 (1981).

[55] Proakis JG, Monolakis DG (2007). Digital signal processing.

[56] Schmickler W, Santos E (2010). Interfacial Electrochemistry.

