# Supplementary material for: Non-equilibrium phase transition at a critical point of human blood
Source: Sci Rep. 2021 Nov 17;11:22398. doi: 10.1038/s41598-021-01909-9 (PMC8599461; doi:10.1038/s41598-021-01909-9)
Supplement: Supplementary file 1 — Supplementary Information. [file 41598_2021_1909_MOESM1_ESM.docx]

Supplementary Materials for

**Non-equilibrium phase transition at a critical point of human blood**

Mariusz A. Pietruszka

*Corresponding author. Email: [mariusz.pietruszka@us.edu.pl](mailto:mariusz.pietruszka@us.edu.pl)

**This PDF file includes:**

Table S1

Figs. S1 to S10

Table S1. Complexity metrics of an isolated droplet of human blood at a critical temperature.

| meas #^*^  (°C) | Applied  magnetic  field (B) | Corrected R/S Hurst exponent | Entropy ChaoShen  (in bits) | Entropy ApEn  Sample | Lyapunov exponent  (Λ) | Spectral exponent  (β) |
| --- | --- | --- | --- | --- | --- | --- |
| 00128  @36.05(5) | 0 | 0.9764564 | 2.52  (3.64) | 0.1526319  0.1203923  *deterministic* | -13.88851 | 1.024(27) |
| 00108  @35.95(5) | 30(5) mT | 0.9306849 | 2.03  (2.93) | 0.1128443  0.0692210  *deterministic* | -12.87866 | 1.463(29) |
| 00149  @36.05(5) | 65(5) mT | 0.8966525 | 1.63  (2.36) | 0.8894393  0.8039913  *random* | -16.08789 | 0.641(25) |

*) Raw data files are available at reasonable request.


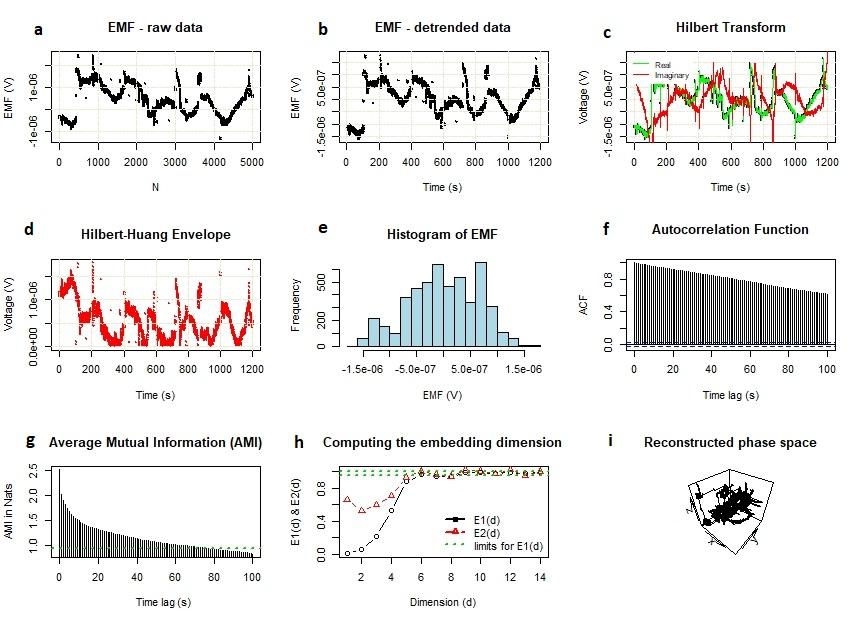


Figure S1 Different complexity measures of an isolated droplet of human blood at temperatures close to 36 °C. Magnetic field $\left| \boldsymbol{B} \right|\boldsymbol{=}0$. (a) Electromotive force (EMF) as a function of the point counter ($N$) (b) detrended data from a (c) Hilbert transform (d) Hilbert-Huang envelope (e) Histogram of EMF (f) Autocorrelation function (g) Average mutual information as a function of the time delay (h) Computing the embedding dimension (i) Reconstructed trajectory of the phase space according to Takens' theorem. Representative charts from 169 measurement series.


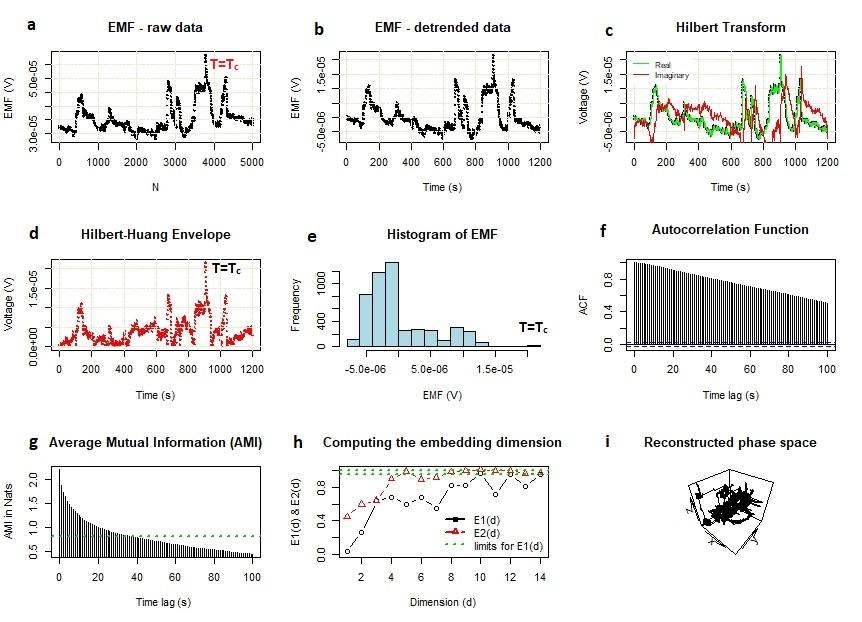


Figure S2 Different complexity measures of an isolated droplet of human blood at temperatures close to 36 ^°^C. Magnetic field $\left| \boldsymbol{B} \right|\boldsymbol{=}30(5)$ mT. The EMF peaks occurred at critical points when the resonance conditions were met (indicated), i.e. where $T=T_{c}$ – charged avalanches appeared; transition to zero resistance is met at $T_{c}$. The further description as for Fig. S1.


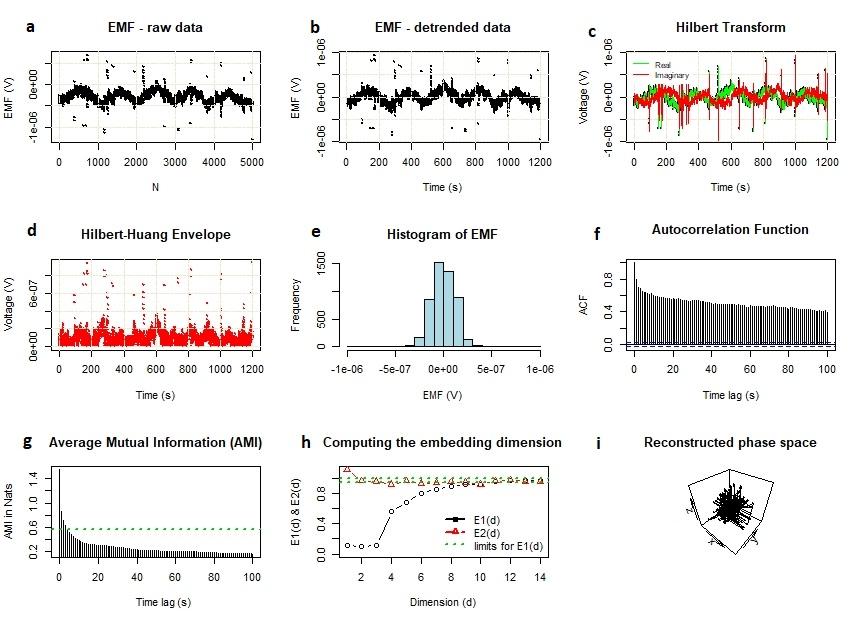


Figure S3 Different complexity measures of an isolated droplet of human blood at a critical temperature. Magnetic field $\left| \boldsymbol{B} \right|\boldsymbol{=}60\left( 5 \right)$ mT (critical field effect). The further description as for Fig. S1.


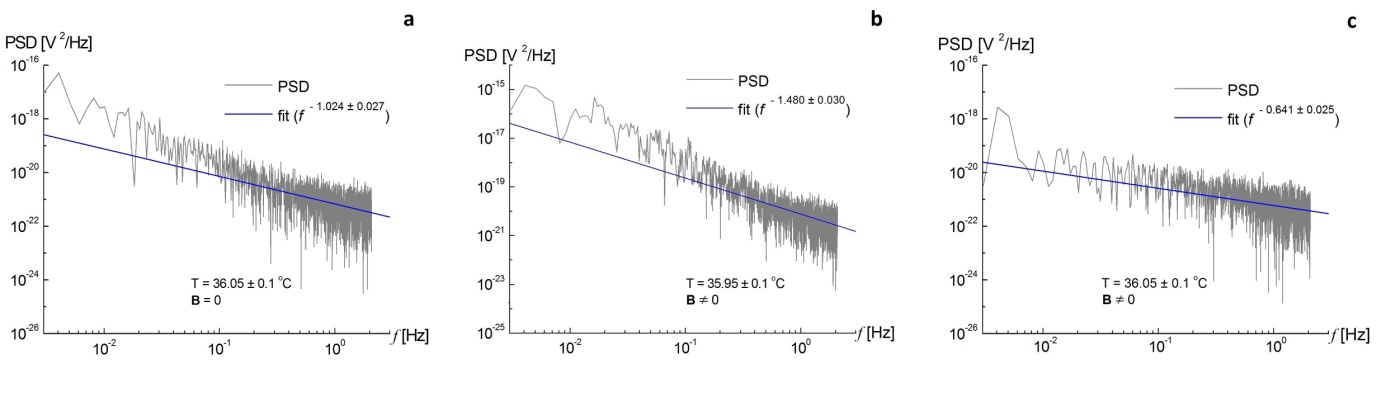


**Figure S4 Power spectral density (PSD) as a function of the frequency *f* at a critical temperature.** Calculated for the EMF that was induced by the ionic fluxes of an isolated droplet of peripheral human blood in double-logarithmic coordinates. (a) Pink noise for $B=\left| \boldsymbol{B} \right|=0$. The actual fit, which resulted in a power law with the spectral exponent $\beta=1.024 (27)$ is indicated by the blue line (decaying as $1/f$). (b) Brown noise, $\beta=1.480(30)$ for $B=30(5)$ mT. (c) Pink/white noise, $\beta=0.641(25)$ for $B= 60(5)$ mT. In (b) and (c) a constant homogeneous magnetic field that was induced by a ferrite toroid was applied.

**
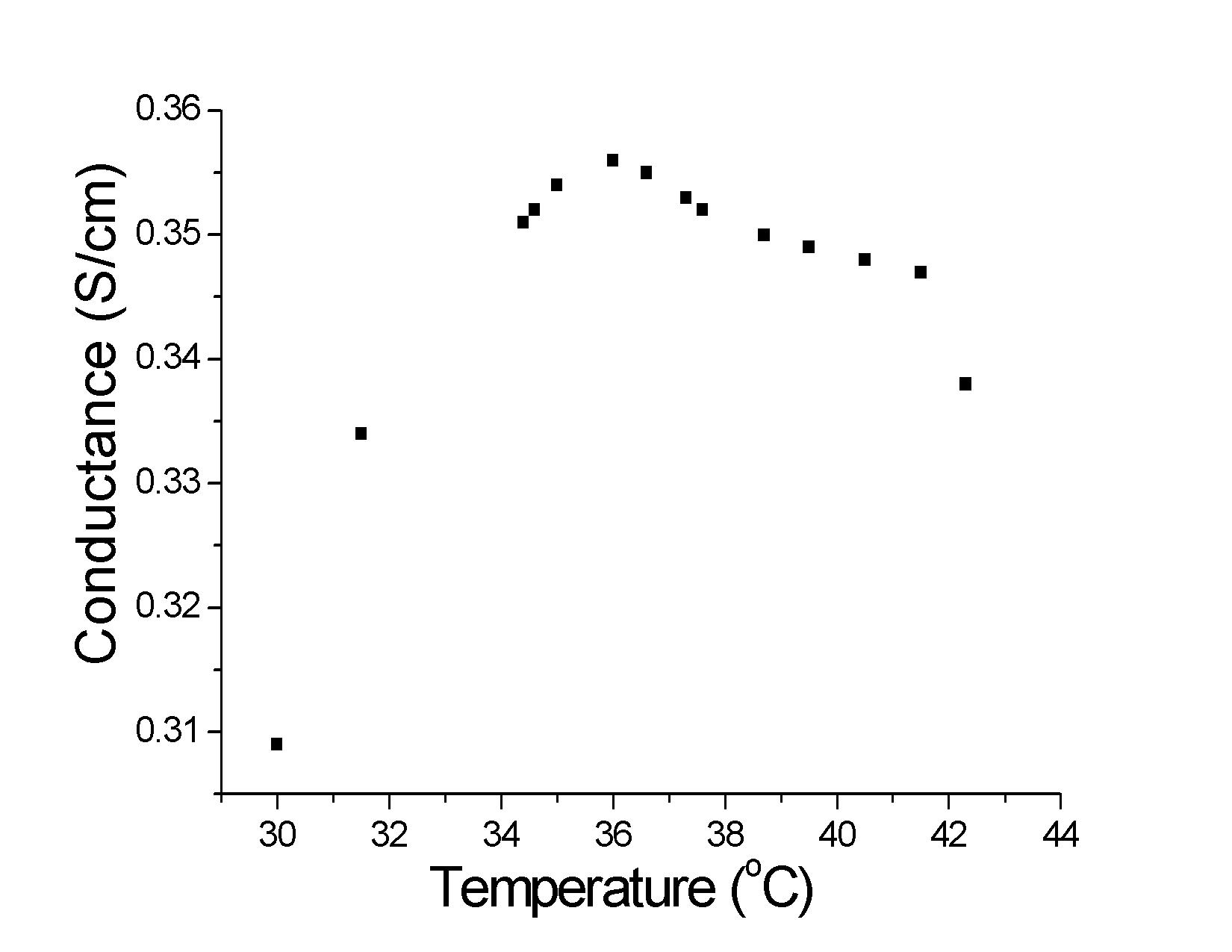
**

**Figure S5 Conductivity of the diluted blood sample.** Note the cut-off temperature at 42^°^C.


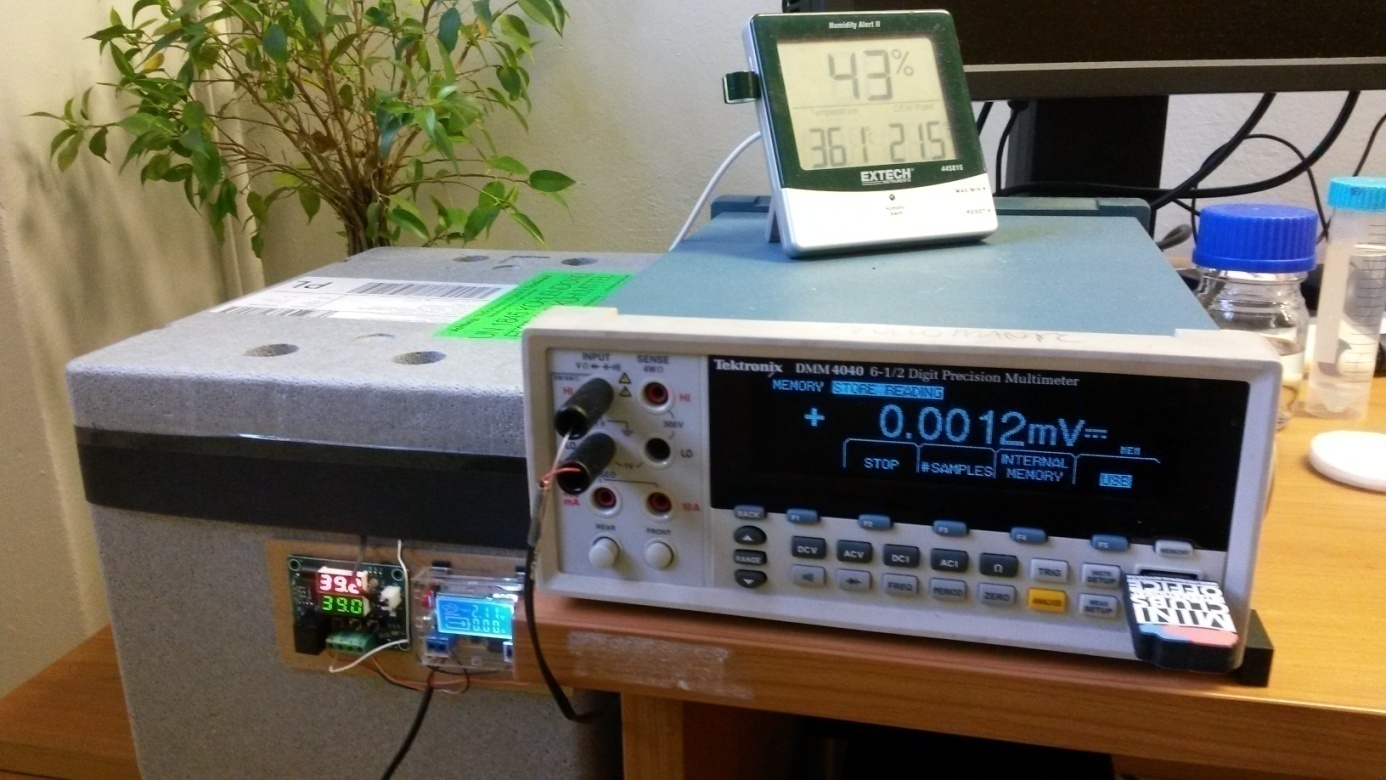


Figure S6 Experimental setup.


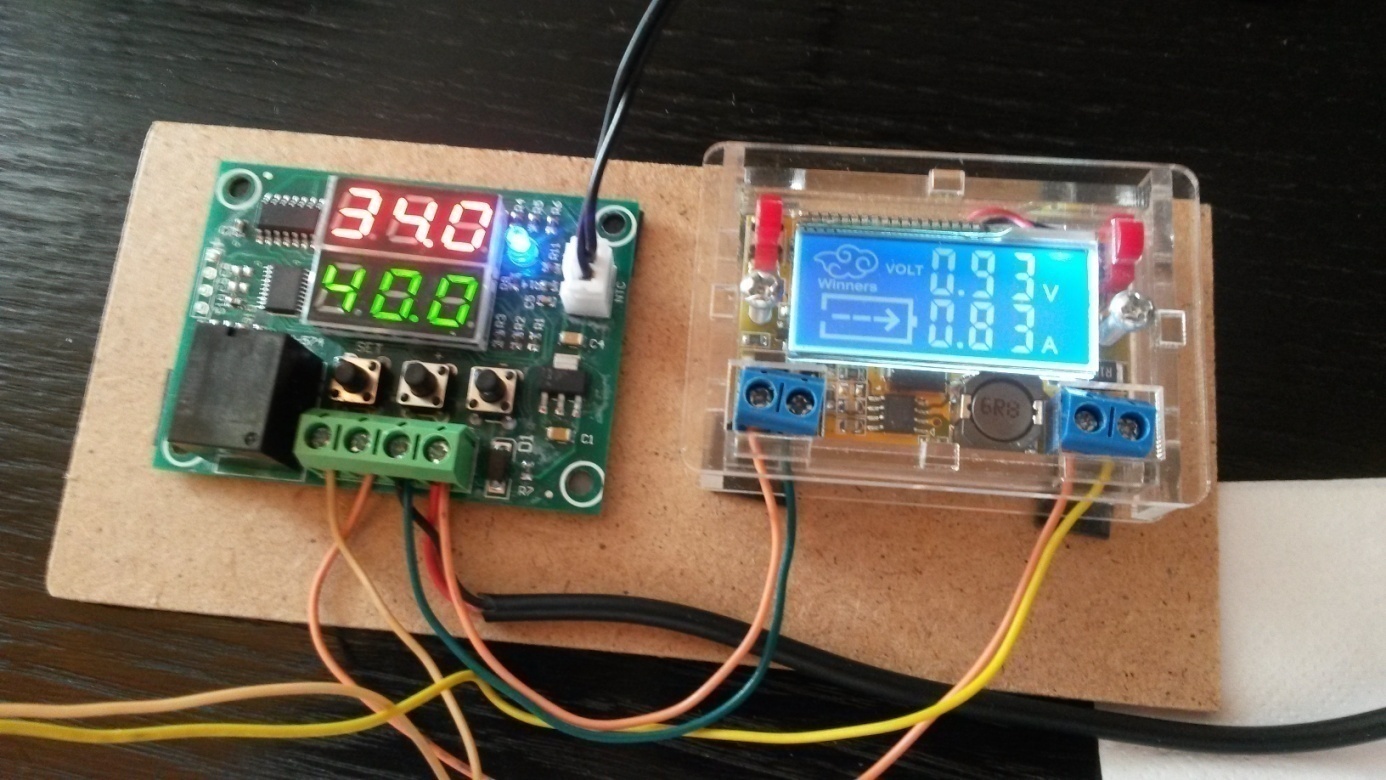


Figure S7 Temperature stabilisation circuit.


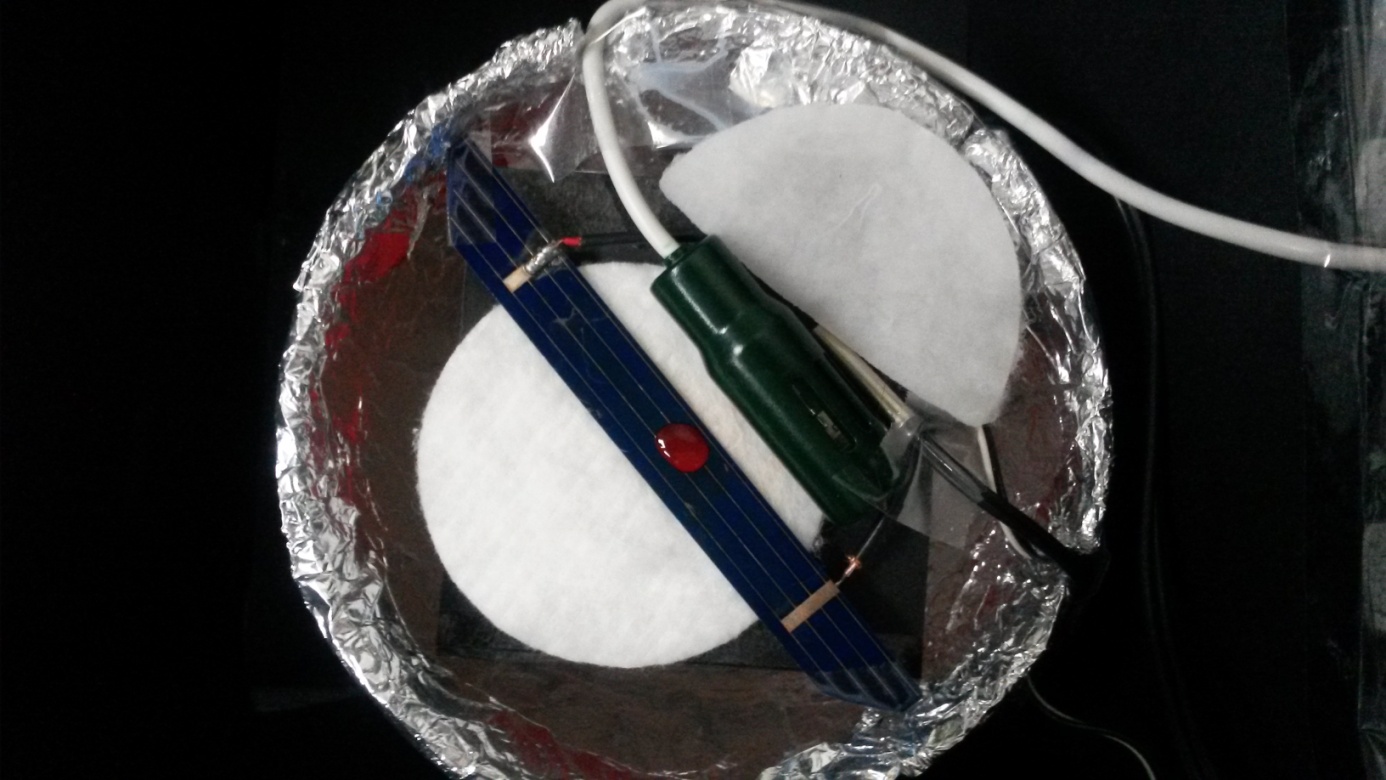


Figure S8 Measuring chamber (open).


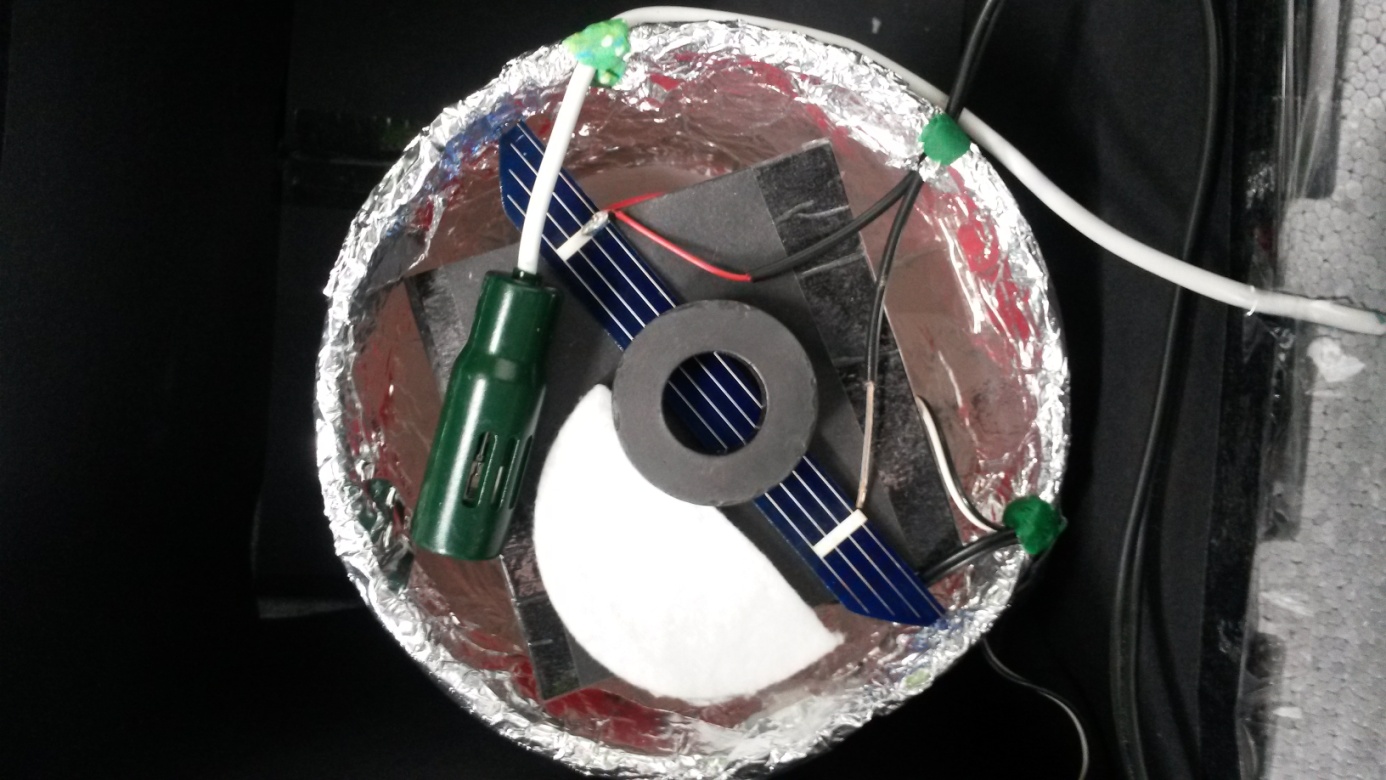


**Figure S9 Measuring chamber with a ferrite magnet.**


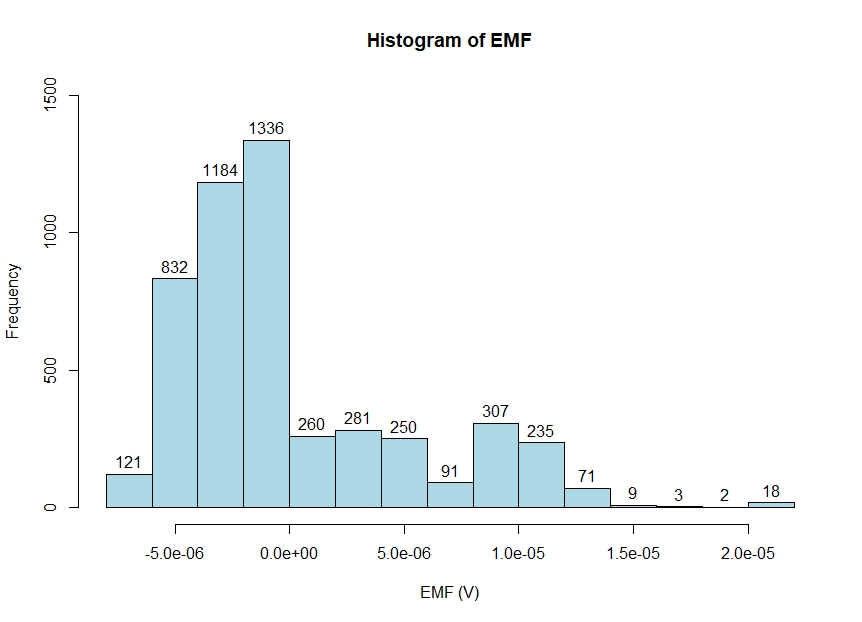


**Figure S10** Histogram of the electromotive force. Note the value of 18 × 2.0 × 10^-5^ V.
